# Total cardiovascular or fatal events in people with type 2 diabetes and cardiovascular risk factors treated with dulaglutide in the REWIND trail: a post hoc analysis

**DOI:** 10.1186/s12933-020-01179-1

**Published:** 2020-11-25

**Authors:** Gilles R. Dagenais, Lars Rydén, Lawrence A. Leiter, Mark Lakshmanan, Leanne Dyal, Jeffrey L. Probstfield, Charles Messan Atisso, Jonathan E. Shaw, Ignacio Conget, William C. Cushman, Patricio Lopez-Jaramillo, Fernando Lanas, Ernesto German Cordona Munoz, Valdis Pirags, Nana Pogosova, Jan Basile, Wayne H. H. Sheu, Theodora Temelkova-Kurktschiev, Peter J. Raubenheimer, Matyas Keltai, Stephanie Hall, Prem Pais, Helen M. Colhoun, Matthew C. Riddle, Hertzel C. Gerstein

**Affiliations:** 1 Clinical Research Center, Laval University, Quebec Heart and Lung Institute, 2725, chemin Ste-Foy, Quebec City, Qc GIV 4G5 Canada; 2 Department of Medicine Solna, Karolinska Institute, Stockholm, Sweden; 3 Li Ka Shing Knowledge Institute, St. Michael’s Hospital, University of Toronto, Toronto, ON Canada; 4 Eli Lilly and Company, Indianapolis, In USA; 5 Population Health Research Institute, McMaster University and Hamilton Health Science, Hamilton, ON Canada; 6 Department of Medicine (Cardiology), University of Washington Medical Centre, Seattle, WA USA; 7 Baker Heart and Diabetes Institute, Melbourne, VIC Australia; 8 Endocrinology and Nutrition Department, Hospital Clinic i Universitari, Barcelona, Spain; 9 Department of Medicine, University of Tennessee Health Science, Memphis, TN USA; 10 Masira Research Institute, Medical School, Universidad de Santander UDES, Bucaramanga, Colombia; 11 Universidad de la Frontera, Temuco, Chile; 12 Universidad de Guadalajara Centro Universitario de Ciencias de la Salud, Guadalajara, Mexico; 13 Latvijas Universitate, Riga, Latvia; 14 National Medical Research Centre of Cardiology, Moscow, Russia; 15 Medical University of South Carolina, Charleston and Ralph H Johnson VA Medical Center, Charleston, SC USA; 16 Endocrinology and Metabolism, Taichung Veterans General Hospital, Taichung, Taiwan; 17 Robert Koch Medical Centre, Sofia, Bulgaria; 18 Department of Medicine, University of Cape Town, Cape Town, South Africa; 19 Semmelweis University, Hungarian Institute of Cardiology, Budapest, Hungary; 20 St John’s Research Institute, Bangalore, India; 21 MRC Institute of Genetics and Molecular Medicine, University of Edinburgh, Edinburgh, UK; 22 Department of Medicine, Oregon and Health Science University, Portland, OR USA

**Keywords:** Type 2 diabetes, Cardiovascular disease, Glucagon like peptide-1 receptor agonists

## Abstract

**Background:**

The Researching cardiovascular Events with a Weekly INcretin in Diabetes (REWIND) double blind randomized trial demonstrated that weekly subcutaneous dulaglutide 1.5 mg, a glucagon like peptide-1 receptor agonist, *versus* matched placebo reduced the first outcome of major adverse cardiovascular event (MACE), cardiovascular death, nonfatal myocardial infarction or nonfatal stroke (594 versus 663 events) in 9901 persons with type 2 diabetes and either chronic cardiovascular disease or risk factors, and followed during 5.4 years. These findings were based on a time-to-first-event analysis and preclude relevant information on the burden of total major events occurring during the trial. This analysis reports on the total cardiovascular or fatal events in the REWIND participants

**Methods:**

We compared the total incidence of MACE or non-cardiovascular deaths, and the total incidence of expanded MACE (MACE, unstable angina, heart failure or revascularization) or non-cardiovascular deaths between participants randomized to dulaglutide and those randomized to placebo. Incidences were expressed as number per 1000 person-years. Hazard ratios (HR) were calculated using the conditional time gap and proportional means models.

**Results:**

Participants had a mean age of 66.2 years, 46.3% were women and 31% had previous cardiovascular disease. During the trial there were 1972 MACE or non-cardiovascular deaths and 3673 expanded MACE or non-cardiovascular deaths. The incidence of total MACE or non-cardiovascular deaths in the dulaglutide and placebo groups was 35.8 and 40.3 per 1000 person-years, respectively [absolute reduction = 4.5 per 1000 person-years; conditional time gap HR 0.90 (95% CI, 0.82–0.98) p = 0.020*,* and proportional means HR 0.89 (95% CI, 0.80–0.98) p = 0.022]. The incidence of total expanded MACE or non-cardiovascular deaths in the dulaglutide and placebo groups was 67.1 and 74.7 per 1000 person-years, respectively [absolute reduction = 7.6 per 1000 person-years; conditional time gap HR 0.93 (95% CI, 0.87–0.99) p = 0.023, and proportional means HR 0.90 (95% CI, 0.82–0.99) p = 0.028].

**Conclusions:**

These findings suggest that weekly subcutaneous dulaglutide reduced total cardiovascular or fatal event burden in people with type 2 diabetes at moderate cardiovascular risk.

*Clinical Trial Registration:* https://www.clinicaltrials.gouv. Unique Identifier NCT01394952).

## Background

People with diabetes continue to be at high risk for cardiovascular (CV) events and premature death despite the use of established cardioprotective therapies. Large randomized placebo-controlled CV outcomes trials have therefore been conducted to assess the effects of glucose-lowering drugs on the hazard of major adverse CV events (MACE) defined as nonfatal myocardial infarction, nonfatal stroke or death from CV causes. Seven such trials using glucagon-like peptide-1 receptor agonists (GLP-1 RAs) have now been published. Four of them reported a reduced hazard of MACE [1–4], and a meta-analysis of these plus three additional trials whose results were neutral [5–7], reported a statistically significant hazard ratio of 0.88 for this outcome [8]. One of these trials was the Researching cardiovascular Events with a Weekly INcretin in Diabetes (REWIND) trial, in which the addition of a once-weekly subcutaneous injection of dulaglutide (1.5 mg) significantly reduced the hazard of MACE by 12% in 9901 individuals with type 2 diabetes with either chronic CV disease or CV risk factors [4]. It also clearly demonstrated similar effect sizes in the subgroups with (31.5% of participants) and without (68.5% of participants) known CV disease.

These 7 CV outcomes trials all reported time-to-occurrence of the first MACE. Limiting the analysis to time-to-the first event precludes relevant clinical information pertaining to the total event burden experienced by participants [9–16]. To assess the impact of dulaglutide on total major events in the REWIND trial, we compared the composite of total number of MACE or non-CV deaths and the composite of total number of expanded MACE (MACE, heart failure, unstable angina or revascularization) or non-CV deaths between participants randomized to the GLP-1 RA and those randomized to placebo.

## Methods

### Trial design and study population

The trial design, population recruitment, baseline characteristics and results have been reported [4, 17, 18]. In brief, REWIND was a randomized, double-blind placebo-controlled trial done at 371 sites in 24 countries. Eligibility criteria included age 50 years and older with established or newly diagnosed type 2 diabetes, a HbA1c ≤ 9.5% on stable doses of up to 2 oral glucose-lowering drugs, and a body mass index (BMI) ≥ 23 kg/m^2^. In addition, people ≥ age 50 had to have CV disease (previous MI, ischemic stroke, revascularization, hospitalization for unstable angina or imaging evidence of myocardial ischemia), those ≥ age 55 had to have myocardial ischemia or coronary, carotid or lower limb arterial stenosis greater than 50%, left ventricular hypertrophy, albuminuria or estimated glomerular filtration rate (eGFR) < 60 mL/min per 1.73 m^2^, and those ≥ age 60 without evidence of CV or renal disease needed to have at least 2 of the following risk factors: hypertension, dyslipidemia, tobacco consumption or abdominal obesity. The main exclusion criteria were: cancer within the previous 5 years, eGFR < 15 mL/min per 1.73 m^2^, severe hypoglycemia in the previous year, liver disease, life expectancy less than 1 year, a CV event within the previous 2 months, or plans for revascularisation. The REWIND protocol was approved by Ethics Committees at all participating sites and all participants provided written informed consent.

### Random assignment and follow-up

REWIND participants were recruited between August 18, 2011 and August 14, 2013. After a 3-week run-in period, eligible participants were randomized to either a weekly subcutaneous injection of dulaglutide 1.5 mg or matched placebo. Follow-up visits were every 3 to 6 months to document any CV disease or other health problems and to give lifestyle guidance. Among the participants randomized to dulaglutide 82.2% took the study medication from randomization until either a primary outcome or final follow-up compared with 83.1% of those randomized to placebo. All hospitalizations were recorded. REWIND was an event-driven trial with a target of at least 1200 confirmed first occurrences of a MACE consisting of CV death, nonfatal MI or nonfatal stroke. The trial was terminated on August 21, 2018 at which point, 1257 people had experienced a confirmed first MACE.

### Events

The analyses for this report focus on the composite of total number of pre-specified MACE or non-CV deaths, and the composite of total number of expanded MACE outcomes (MACE, heart failure requiring hospitalization or urgent medical visit for treatment, unstable angina requiring hospitalization for treatment, or coronary, carotid or peripheral revascularization) or non-CV deaths from any cause. Various combinations of CV or fatal events were also analyzed. All of the events reported here were adjudicated by an independent committee of physicians blinded to the study medication and used pre-specified definitions [4].

### Statistical analyses

Categorical variables were summarized by counts and proportions, and continuous variables by means and standard deviations (SD). Trends in proportions were assessed using the Cochrane-Armitage trend test, and trends in means were assessed using simple linear regression. Analyses were performed separately for each composite event and focused on the total number of events during follow-up, and not the number of people with one or more events. Therefore, an individual who experienced a heart failure event, followed by a MACE, and later on by death from pneumonia would be counted as having had 3 events.

Incidence rates of events were estimated as the number of events per 1000 person-years (py) of follow-up. Hazard ratios (HR) and 95% confidence intervals (CI) for total MACE or non-CV deaths and total expanded MACE or non-CV deaths were estimated using both the conditional gap time model [19] and the proportional means model [20]. Cox proportional hazards models were used to estimate the HR and 95% CI of first outcomes. All analyses were done using SAS (version 9.4).

## Results

### Baseline characteristics

The REWIND trial randomly assigned 9901 people, including 4589 (46.3%) women and 5312 (53.7%) men of mean (SD) age 66.2 (6.5) years to the addition of weekly subcutaneous injections of either dulaglutide 1.5 mg or placebo. Detailed baseline characteristics according to randomized treatment group have already been published [4]. Baseline characteristics in people who did and did not experience one or more MACE or non-CV deaths during follow-up are presented in Table 1. Numbers of events experienced during follow-up were statistically correlated with age, male sex, white background, tobacco use, prior CV disease, heart failure, diabetes duration, systolic blood pressure, HbA1c, use of beta blockers, blood pressure drugs (other than renin-angiotensin system modulators), antiplatelet drugs, sulfonylureas and insulin, and were inversely related to eGFR, and metformin use.

**Table 1:** Distribution of number MACE or non-CV deaths according to baseline characteristics

	Participants	Number of events per participant			P (trend)
		0	1	≥ 2	
Randomized	9901	8233	1425	243	
Mean age (years)	9901	65.8 (6.3)	68.1 (7.3)	67.9 (8.0)	< 0.0001
Female	4589	3972 (48.2)	522 (36.6)	95 (39.1)	< 0.0001
White	7498	6207 (75.4)	1096 (76.9)	195 (80.3)	0.044
Tobacco consumption	1407	1113 (13.5)	254 (17.8)	40 (16.5)	< 0.0001
Prior cardiovascular disease	3114	2362 (28.7)	614 (43.1)	138 (56.8)	< 0.0001
Hypertension	9224	7658 (93.0)	1338 (93.9)	228 (93.8)	0.23
Prior heart failure	853	626 (7.6)	184 (12.9)	43 (17.7)	< 0.0001
Mean diabetes duration (years)	9901	10.3 (7.04)	11.4 (7.91)	11.8 (8.67)	< 0.0001
Mean BMI (kg/m^2^)	9900	32.4 (5.7)	32.0 (5.78)	32.6 (6.12)	0.16
Mean SBP (mm Hg)	9901	137 (16.5)	140 (18.0)	139 (17.7)	< 0.0001
Mean DBP (mm Hg)	9901	78.5 (9.7)	78.6 (10.3)	77.2 (10.6)	0.463
Mean HbA1c	9876	7.3 (1.0)	7.4 (1.1)	7.4 (1.0)	0.0062
Mean eGFR (mL/min per 1.73 m^2^)	9640	77.9 (22.3)	71.7 (24.0)	70.7 (23.8)	< 0.0001
Mean LDL (mmol/L)	9590	2.55 (0.97)	2.64 (1.00)	2.48 (0.96)	0.092
Medications					
Metformin	8037	6737 (81.8)	1111 (78.0)	189 (77.8)	0.0004
Sulfonylurea	4552	3740 (45.4)	700 (49.1)	112 (46.1)	0.0430
Insulin	2363	1889 (22.9)	409 (28.7)	65 (26.8)	< 0.0001
DPP4i	564	484 (5.9)	64 (4.5)	16 (6.6)	0.220
Thiazolidinedione	168	150 (1.8)	14 (1.0)	4 (1.7)	0.073
Other glucose lowering drugs	32	28 (0.3)	4 (0.3)	0	0.39
ACEI/ARB	8068	6700 (81.4)	1166 (81.8)	202 (83.1)	0.47
Beta blocker	4512	3628 (44.1)	737 (51.7)	147 (60.5)	< 0.0001
Other BP drug	5599	4568 (55.5)	871 (61.1)	160 (65.8)	< 0.0001
Statin	6547	5450 (66.2)	907 (63.7)	190 (78.2)	0.27
Fibrate	898	756 (9.2)	119 (8.4)	23 (9.5)	0.52
Antiplatelet	5342	4365 (53.0)	812 (57.0)	165 (67.9)	< 0.0001

Data are shown as N (%) or Mean (SD); BMI, body mass index; SBP, systolic blood pressure; DBP, diastolic blood pressure; HbA1c, glycated hemoglobin A1c; eGFR, estimated glomerular filtration rate; LDL, low density lipoprotein; DPP4i, dipeptidyl-peptidase-4 inhibitor; ACEI/ARB, angiotensin converting enzyme inhibitor/angiotensin receptor blocker

### Events during follow-up

During a median follow-up period of 5.4 years (IQR 5.1–5.9), 1257 of the 9901 (12.7%) participants experienced at least one MACE, 1918 (19.4%) experienced at least one expanded MACE (Additional file 1: Table S1), and 465 (4.7%) died from non-CV causes.

By the end of follow-up there were a total of 932 MACE or non-CV deaths in dulaglutide group participants (35.8 per 1000 py) and 1040 such outcomes (40.3 per 1000 py) in placebo group participants (Table 2). The total number of MACE or non-CV deaths was, therefore, reduced by an absolute amount of 4.5 per 1000 py in the dulaglutide treatment group. This was reflected in a 10% reduced hazard using the conditional time gap model [HR 0.90 (95% CI, 0.82–0.98) p = 0.020] and an 11% reduced hazard using the proportional means model [HR 0.89 (95% CI, 080–0.98) p = 0.022]. Similar effects were noted within subgroups (Additional file 1: Table S2) defined by age, sex, prior CV disease, HbA1c levels, and use of statins, renin-angiotensin drugs or metformin (interaction P values all ≥ 0.15).

**Table 2:** Incidences and hazard ratios of the composites of total CV events or non-CV deaths

Composite event	Dulaglutide group	Placebo group	Events avoided^a^	Conditional time gap model		Proportional means model	
	N (/1000py)	N (/1000py)	N (N/1000py)	HR (95% CI)	p	HR (95% CI)	P
MACE or non-CV death	932 (35.8)	1040 (40.3)	108 (4.5)	0.90 (0.82–0.98)	0.020	0.89 (0.80–0.98)	0.022
MACE, UA or non-CV death	1034 (39.7)	1123 (43.5)	89 (3.8)	0.92 (0.85–1.00)	0.052	0.91 (0.83–1.01)	0.066
MACE, HF or non-CV death	1195 (45.9)	1372 (53.2)	177 (7.3)	0.91 (0.84–0.99)	0.021	0.86 (0.78–0.96)	0.006
MACE, Revasc or non-CV death	1399 (53.8)	1519 (58.9)	120 (5.1)	0.92 (0.86–0.99)	0.030	0.91 (0.83–1.01)	0.0632
MACE, UA, HF, Revasc or non-CV death	1747 (67.1)	1926 (74.7)	179 (7.6)	0.93 (0.87–0.99)	0.023	0.90 (0.82–0.99)	0.028

^a^Difference in numbers (and rate) of outcomes in the dulaglutide group versus the placebo group; N, number of outcomes; py, person-year; MACE, major adverse cardiovascular events; CV, cardiovascular; UA, unstable angina; HF, heart failure; Revasc, revascularization

**Fig. 1:**
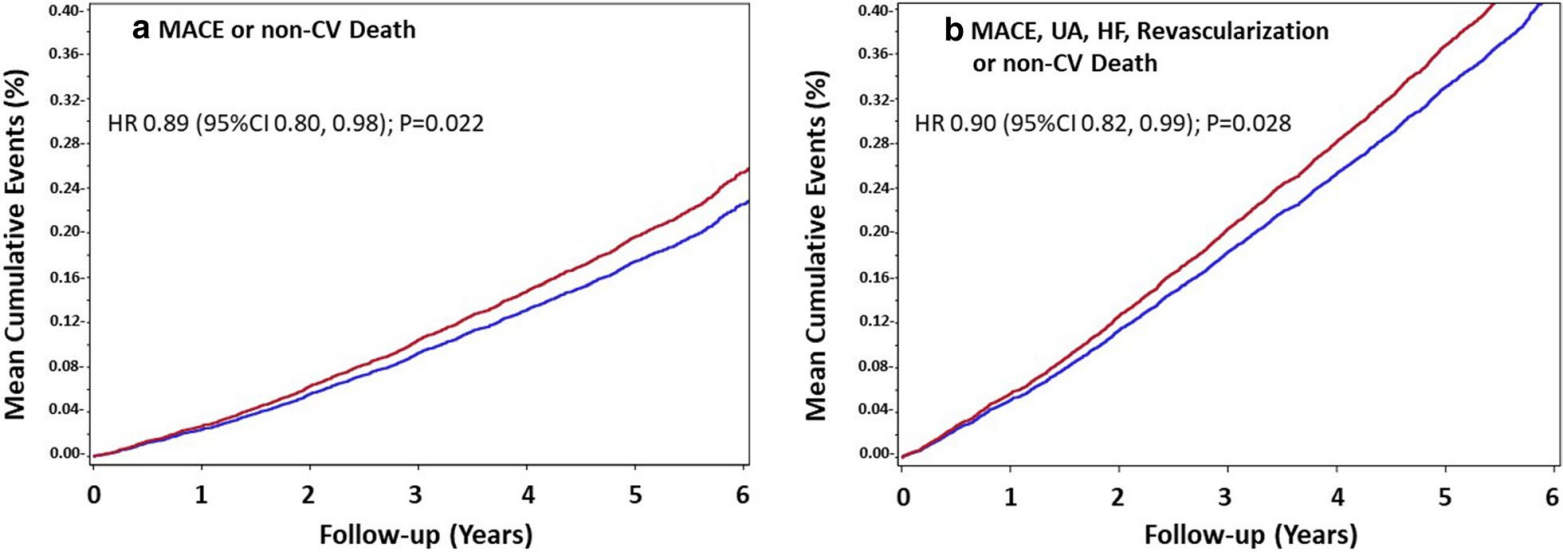
Cumulative incidence of the composite of total MACE or non-CV deaths (**a**) and the composite of total expanded MACE or non-CV deaths (**b**) in the dulaglutide group (blue line) and the placebo group (red line) during the follow-up. The hazard ratios (HR) are from the proportional means model

During the same period there were a total of 1747 expanded MACE or non-CV deaths in dulaglutide group participants (67.1 per 1000 py) and 1926 such outcomes (74.7 per 1000 py) in placebo group participants. The total number of MACE or non-CV deaths was, therefore, reduced by an absolute amount of 7.6 per 1000 py in the dulaglutide treatment group. This was reflected in a 7% reduced hazard using the conditional time gap model [HR 0.93(95% CI (0.87–0.99) p = 0.023] and a 10% reduced hazard using the proportional means model [HR 0.90 (95% CI 0.82–0.99) p = 0.028]. The mean cumulative event curves for both composite outcomes began to diverge after the first year and continued diverging until the completion of the study showing a lower rate of outcomes in participants randomized to dulaglutide than in those randomized to placebo (Fig. 1). Similar treatment group differences were noted for the composite of total MACE, unstable angina or death, total MACE, heart failure or death, and total MACE, revascularization or death (Table 2).

As noted in the Tables 3 and Additional file 1: Table S3, there were 1668 first MACE or non-CV deaths, comprising 789 in the dulaglutide group and 879 in the placebo group, [HR 0.88 (95% CI, 0.80–0.97) p = 0.011]. A similar pattern was seen for the 2286 first expanded MACE or non-CV deaths, that included 1097 in the dulaglutide group and 1189 in the placebo group, [HR 0.91 (95% CI, 0.84–0.99) p = 0.024].

**Table 3:** Estimated effects of dulaglutide on the first composite outcome

Composite event	Dulaglutide group	Placebo group	People spared^a^	Cox model	
	N (N/1000py)	N (N/1000py)	N (N/1000py)	HR (95% CI)	p
MACE or non-CV death	789 (31.2)	879 (35.3)	90 (4.1)	0.88 (0.80, 0.97)	0.011
MACE, UA, or non-CV death	859 (34.3)	933 (37.8)	74 (3.5)	0.91 (0.83, 0.99)	0.041
MACE, HF or non-CV death	880 (35.3)	983 (40.1)	103 (4.8)	0.88 (0.80, 0.96)	0.0054
MACE, Revasc, or non-CV death	1002 (40.8)	1090 (44.9)	88 (4.1)	0.91 (0.83, 0.99)	0.028
MACE, UA, HF, Revasc, or non-CV death	1097 (45.3)	1189 (49.8)	92 (4.5)	0.91 (0.84, 0.99)	0.024

^a^Difference in numbers (and rate) of people experiencing a first composite event in the dulaglutide group versus the placebo group; CV, cardiovascular; py, person-year; MACE, major adverse cardiovascular events; UA, unstable angina; HF, heart failure; Revasc, revascularization

## Discussion

In this post hoc analysis of data from the REWIND trial, participants randomized to a once-weekly subcutaneous injection of dulaglutide 1.5 mg had a reduced total number of CV or fatal outcomes in addition to the number of first CV or fatal events compared with participants randomized to placebo. Moreover, the reduction in absolute numbers of events was greater for expanded MACE or non-CV deaths than for MACE or non-CV deaths in the dulaglutide treatment group.

The pre-specified primary analyses of all CV outcomes trials with GLP-1 receptor agonists, including REWIND, focused on estimating a participant’s risk of experiencing the first primary CV outcome, MACE after randomization. The reported HRs therefore reflect differences in the time from randomization to the first outcome in people who were randomly assigned to the active *versus* control therapy. The previously reported HR of 0.88 for dulaglutide *versus* placebo in the REWIND trial means that on average at any time-point after randomization, people who had been randomly assigned to dulaglutide were 12% less likely to have experienced a first post-randomization MACE than people who had been randomized to placebo [4]. Although this provides important information regarding the therapeutic effect of dulaglutide, patients and health care providers are also interested in knowing the total burden of serious CV events, and deaths from any cause in participants treated with dulaglutide. The estimated HR of about 0.9 for the burden of total MACE or non-CV death therefore focuses on events and not participants. It means that at any time-point after randomization, there were 10% fewer events in people randomly assigned to dulaglutide *versus* placebo. In the participants with a placebo-group MACE or non-CV death rate of 40.3 per 1000 py, this 10% difference would be consistent with 108 fewer MACE or non-CV deaths, and 179 fewer expanded MACE or non-CV deaths (with a placebo rate of 74.7 per 1000 py) in the dulaglutide group during the 5.4-year follow-up. If the results of this post hoc analysis are replicated, we hypothesize that the number of events avoided would be higher in individuals with event rates higher than those reported in Table 2.

The inclusion of non-CV death within the primary analyses presented here represents a conservative approach to estimating the effect of dulaglutide on the overall burden of CV disease as well as on the time to the first occurrence. First, death from any cause is clearly relevant to both participants and providers. Second, including all-cause deaths *versus* just CV deaths eliminates any misclassification of the cause of death (i.e. CV or non-CV death). Finally, it eliminates the possibility that a benefit of the drug on event burden would be negated by an increase in death, also referred to as the competing risk of death [21]. Whereas some investigators addressed this possibility in sensitivity analyses [13–15], we and others have integrated all-cause death into the main analyses of event burden [16]. Notably, the estimated HR of 0.88 for the time to the occurrence of the first MACE outcome or non-CV death was identical to the previously reported HR for the first occurrence of MACE alone [4]. However, the addition of non-CV deaths increases the event rates in both treatment groups compared with MACE alone and hence the number of people spared from experiencing an event. This is reflected in the absolute risk reduction which was 3.1 per 1000 py for the first occurrence of a MACE as previously reported (594 *versus* 663 events) [4], and 4.1 per 1000 py for the first occurrence of a MACE or non-CV death as noted in Additional file 1: Table S3 (789 versus 879 events).

The finding of a reduced burden of CV events in the REWIND trial is similar to the results of an analysis of event burden from the Liraglutide Effect and Action in Diabetes Evaluation of cardiovascular Results (LEADER) trial of another GLP-1 RA. In this analysis liraglutide was associated with a reduction of total (first and recurrent) MACE by 16% and of total CV events by 13% in 9340 participants followed for a median of 3.8 years [15]. Notably, inclusion of non-CV deaths in a sensitivity analysis did not alter their findings. The higher outcome reduction rates in the LEADER trial than in the REWIND mostly result in a higher risk population; indeed, 83% of the LEADER enrolled participants had a chronic CV disease compared with 31% of the REWIND enrolled participants.

Although the exact mechanism of the benefit of dulaglutide or other GLP-1 receptor agonists on CV disease reduction is unknown, possibilities include a decrease of risk factors such as high HbA1c, LDL-cholesterol, blood pressure and bodyweight as well as potential effects on the atherothrombotic process by improving endothelial and platelet functions [22] and attenuating the arterial plaque progression by reducing inflammation and vascular tone [23]. The fact that other GLP-1 receptor agonists such as liraglutide and semaglutide have qualitatively similar effects on CV events, metabolic and other indices overall and within sub-groups is consistent with general “class effect” of these drugs [24–26].

## Limitations

These analyses are limited by the fact that they were not pre-specified and causal conclusions should not be drawn. Strengths include the 5.4-year median follow-up period, the large number of participants from many countries with well characterized baseline clinical findings, the high proportion of enrolled women, and the inclusion of all deaths in the composite event as a measure of total event burden.

## Conclusions

These findings suggest that dulaglutide reduced the event burden of CV or fatal outcomes in a population at moderate CV risk. The absolute reduction was greatest for the composite outcome that included an expanded definition of a MACE. Our study supports the clinical implication of assessing all CV events or deaths in addition to the first manifestation of the primary outcome of randomized trials to capture the impact of the pharmacological intervention on the burden of disease.

## Supplementary information

** Additional file 1: Table S1.** Number of Participants who Experienced Varying Numbers of CV Events. **Table S2.** Effect of dulaglutide on Total MACE or non-Cardiovascular Death in subgroups. **Table S3.** Number of Participants who Experienced Varying Numbers of CV Events or on non CV deaths.

## Data Availability

All the data for the present report came from the Population Health Institute (PHRI) in Hamilton, Canada. PHRI did also all data analysis for the REWIND trial. The REWIND main trial data are in references 4, 17 and 18. The REWIND data sharing policy is described in the Additional file 1.

## Abbreviations

ACEI: Angiotensin converting enzyme inhibitor
ARB: Angiotensin receptor blocker
BMI: Body mass index
BP: Blood pressure
CI: Confidence interval
CV: Cardiovascular
DBP: Diastolic blood pressure
DPP4i: Dipeptidyl-peptidase-4 inhibitor
eGFR: Estimated glomerular filtration rate
GLP-1: Glucagon-like peptide-1
HbA1c: Glycated hemoglobin A1c
HF: Heart failure
HR: Hazard ratio
LDL: Low density lipoprotein
MACE: Major adverse cardiovascular event
MI: Myocardial infarction
py: Person-year
Revasc: Revascularization
SBP: Systolic blood pressure
UA: Unstable angina
