# Supplementary material for: Total cardiovascular or fatal events in people with type 2 diabetes and cardiovascular risk factors treated with dulaglutide in the REWIND trail: a post hoc analysis
Source: Cardiovasc Diabetol. 2020 Nov 25;19:199. doi: 10.1186/s12933-020-01179-1 (PMC7690176; doi:10.1186/s12933-020-01179-1)
Supplement: Supplementary file 1 — Additional file 1: Table S1. Number of Participants who Experienced Varying Numbers of CV Events. Table S2. Effect of dulaglutide on Total MACE or non-Cardiovascular Death in subgroups. Table S3. Number of Participants who Experienced Varying Numbers of CV Events or on non CV deaths. [file 12933_2020_1179_MOESM1_ESM.docx]

**Supplemental Information**

**Page**

**Table S1** Number of Participants who Experienced Varying Numbers of CV Events 2

**Table S2** Effect of dulaglutide on Total MACE or non-Cardiovascular Death 3

in Subgroups

**Table S3** Number of Participants who Experienced Varying Numbers of CV Events

or non-CV Deaths 4

**Data Sharing Policy** 5

| **Table S1 Number of participants who experienced varying numbers of CV events** | | | | |
| --- | --- | --- | --- | --- |
| **Outcome** | **Number of participants** | | | |
|  | **≥ 1 Event** | **0 Event** | **1 Event** | **≥ 2 Events** |
| **MACE** | 1257 | 8644 | 1061 | 196 |
| Dulaglutide (N= 4949) | 594 | 4355 | 503 | 91 |
| Placebo (N = 4952) | 663 | 4289 | 558 | 105 |
| **MACE or UA** | 1386 | 8515 | 1148 | 238 |
| Dulaglutide (N= 4949) | 666 | 4283 | 553 | 113 |
| Placebo (N = 4952) | 720 | 4232 | 595 | 125 |
| **MACE or HF** | 1481 | 8420 | 1110 | 371 |
| Dulaglutide (N= 4949) | 699 | 4250 | 526 | 173 |
| Placebo (N = 4952) | 782 | 4170 | 584 | 198 |
| **MACE or Revascularization** | 1695 | 8206 | 1224 | 471 |
| Dulaglutide (N= 4949) | 811 | 4138 | 582 | 229 |
| Placebo (N = 4952) | 884 | 4068 | 642 | 242 |
| **MACE, UA, HF, or Revascularization** | 1918 | 7983 | 1203 | 715 |
| Dulaglutide (N= 4949) | 921 | 4028 | 577 | 344 |
| Placebo (N = 4952) | 997 | 3955 | 626 | 371 |
| *CV* cardiovascular, *MACE* major adverse cardiovascular events, *N* number of participants, *UA* unstable angina, *HF* heart failure | | | | |

| **Table S2: Effect of Dulaglutide on Total MACE or non-Cardiovascular Death in Subgroups** | | | | | | | | |
| --- | --- | --- | --- | --- | --- | --- | --- | --- |
| **Subgroup** | **Dulaglutide** | | **Placebo** | | **Conditional Time Gap Model** | | **Proportional Means Model** | |
|  | **Events** | **Incidence (N/1000py) py)** | **Events** | **Incidence (N/1000 py)** | **HR (95%CI)** | **P*** | **HR (95%CI)** | **P*** |
| **Overall** | 4949 | 932 (35.8) | 4952 | 1040 (40.3) | 0.90 (0.82, 0.98) | N/A | 0.89 (0.80, 0.98) | N/A |
| **Age ≥ 66** | 2314 | 544 (45.6) | 2350 | 618 (51.6) | 0.90 (0.81, 1.02) | 0.98 | 0.88 (0.77, 1.01) | 0.86 |
| **Age < 66** | 2635 | 388 (27.5) | 2602 | 422 (30.6) | 0.91 (0.79, 1.04) |  | 0.90 (0.77, 1.05) |  |
| **Males** | 2643 | 582 (42.2) | 2669 | 654 (47.4) | 0.90 (0.80, 1.00) | 0.92 | 0.89 (0.78, 1.01) | 1.00 |
| **Females** | 2306 | 350 (28.6) | 2283 | 386 (32.2) | 0.92 (0.79, 1.06) |  | 0.89 (0.75, 1.05) |  |
| **Prior CVD** | 1560 | 437 (54.6) | 1554 | 496 (62.7) | 0.89 (0.78, 1.01) | 0.972 | 0.87 (0.75, 1.01) | 0.86 |
| **No Prior CVD** | 3093 | 439 (26.6) | 3128 | 495 (30.0) | 0.89 (0.78, 1.02) |  | 0.89 (0.77, 1.02) |  |
| **HbA1c ≥ 7.2** | 2610 | 506 (37.1) | 2603 | 574 (42.5) | 0.89 (0.79, 1.00) | 0.84 | 0.87 (0.76, 1.00) | 0.832 |
| **HbA1c < 7.2** | 2329 | 420 (34.0) | 2334 | 465 (38.1) | 0.91 (0.79, 1.03) |  | 0.89 (0.77, 1.04) |  |
| **Statin** | 3279 | 622 (35.9) | 3268 | 719 (42.1) | 0.86 (0.77, 0.96) | 0.15 | 0.85 (0.75, 0.97) | 0.23 |
| **No Statin** | 1670 | 310 (35.7) | 1684 | 321 (36.8) | 0.98 (0.84, 1.15) |  | 0.97 (0.82, 1.14) |  |
| **ACEI or ARB** | 4009 | 761 (36.1) | 4059 | 859 (40.6) | 0.90 (0.81, 0.99) | 0.89 | 0.89 (0.79, 1.00) | 0.95 |
| **No ACEI or ARB** | 940 | 171 (34.6) | 893 | 181 (39.2) | 0.91 (0.74, 1.12) |  | 0.88 (0.70, 1.12) |  |
| **Baseline Meformin** | 4022 | 728 (34.3) | 4015 | 807 (38.4) | 0.90 (0.82, 1.00) | 0.89 | 0.89 (0.80, 1.00) | 0.85 |
| **No Baseline Metformin** | 927 | 204 (42.6) | 937 | 233 (48.7) | 0.88 (0.73, 1.06) |  | 0.87 (0.70, 1.08) |  |
| *P for interaction *MACE* major adverse cardiovascular events, *py*, person-years, *CVD* cardiovascular disease, *HbA1c* glycated hemoglobin A1c, *ACEI* angiotensin converting enzyme inhibitor, *ARB* angiotensin receptor blocker | | | | | | | | |

| **Table S3 Number of participants who experienced varying numbers of CV events or non-CV deaths** | | | | |
| --- | --- | --- | --- | --- |
| **Outcome** | **Number of participants** | | | |
|  | **≥ 1 Event** | **0 Events** | **1 Event** | **≥ 2 Events** |
| **MACE or non-CV death** | 1668 | 8233 | 1425 | 243 |
| Dulaglutide (N= 4949) | 789 | 4160 | 680 | 109 |
| Placebo (N = 4952) | 879 | 4073 | 745 | 134 |
| **MACE, UA, or non-CV death** | 1792 | 8109 | 1502 | 290 |
| Dulaglutide (N= 4949) | 859 | 4090 | 726 | 133 |
| Placebo (N = 4952) | 933 | 4019 | 776 | 157 |
| **MACE, HF or non-CV death** | 1863 | 8038 | 1428 | 435 |
| Dulaglutide (N= 4949) | 880 | 4069 | 680 | 200 |
| Placebo (N = 4952) | 983 | 3969 | 748 | 235 |
| **MACE, revascularization or non-CV death** | 2092 | 7809 | 1570 | 522 |
| Dulaglutide (N= 4949) | 1002 | 3947 | 756 | 246 |
| Placebo (N = 4952) | 1090 | 3862 | 814 | 276 |
| **MACE, UA, HF, revascularization or non-CV death** | 2286 | 7615 | 1505 | 781 |
| Dulaglutide (N= 4949) | 1097 | 3852 | 726 | 371 |
| Placebo (N = 4952) | 1189 | 3763 | 779 | 410 |
| *MACE* major adverse cardiovascular events, *CV* cardiovascular, *N* number of participants, *UA* unstable angina *HF* heart failure | | | | |

**Population Health Research Institute (PHRI) Data Sharing Policy**

Effective Date: July 27, 2018

Data will be disclosed only upon request and approval of the proposed use of the data by a review committee. Membership in the review committee will be determined by the executive leadership of the study. Generally only those requests made by a journal's statistician regarding the data related to the results of the publication will be considered unless the review committee sees high merit in other requests. The following principles will apply to requests:

1. The review committee will have established criteria to review the request to ensure that patient privacy and rights, and PHRI data and research integrity can be maintained with the sharing of the data. This includes (but is not limited to) demonstrated competence related to data security and data analysis by the investigator requesting access. The review committee will also ensure that provision of data to external parties does not contravene any prior agreement with any other parties.
2. PHRI will make individual participant data available, including data dictionaries, within the requirements and/or restrictions of REB/IRB and subject to the conditions set forth in the consent forms of the study. Data provided will be limited to data which underlies the results in the main publication after de-identification. Any analyses and publications should be reviewed and approved by PHRI before publication to ensure that the analyses are accurate and that the publication is not misleading.
3. The study protocol and the statistical analysis plan for analysis of the primary results will be shared.
4. For those requests that originate from concerns expressed by the journal about the data or statistical analyses, the data will be available to the journal statisticians in a timely manner.
5. Data can be disclosed for all other requests from 2 years after the main paper is published plus 6 additional months for each year of study conduct. However, there will be a maximum of 7 years to the time limit restriction.
6. Data will be shared to achieve the objective in the approved proposal with no additional analysis permitted without approval. Only proposals for analyses that do not compete with ongoing analyses or analyses proposed by study investigators will be approved.
7. Data will be made available by one of the following mechanisms. 1) The Statistics Department at PHRI can perform the analysis in accordance with the SAP provided by the investigator and under his/her supervision or 2) Arrangement can be made to transfer the data to a secure location using a process that has been verified by the Director of Statistics at PHRI.
8. Every proposal must identify and provide funding sufficient to defray the cost of data preparation, storage, transfer and analysis for the organization incurring these costs (this may include studies not fully funded from external sources i.e. industry or peer review grants). On occasions where the new analyses proposed are of sufficient scientific interest to PHRI, then a collaborative agreement for joint analyses and publication can be developed and charges may be reduced.
9. The data will be provided for a specified time limit that can allow completion of the analyses that is proposed. At the end of the proposed analyses, the requesting party undertakes to return or destroy the data base provided and provide written documentation of this.

**References**

1. Taichman DB, Sahni, P, Pinborg A et al.Data Sharing Statements for Clinical Trials - A Requirement of the International Committee of Medical Journal Editors. N Engl J Med 2017; 376: 23:2277-9.
2. International Consortium of Investigators for Fairness in Trial Data. Devereaux PJ, Guyatt G, Gerstein H, Connolly S, Yusuf S. Toward Fairness in Data Sharing. N Engl J Med 2016;375:405-7.

Supplemental Endorsers of Article listed in 2
